# Association of psychological factors with limb disability in patients with cervical radiculopathy: comparison with carpal tunnel syndrome

**DOI:** 10.1186/s12891-022-05593-2

**Published:** 2022-07-14

**Authors:** Mahla Daliri B.O., Hamidreza Mazloum Khorasani, Neda Daliri Beirak Olia, Amin Azhari, Mohammadtaghi Shakeri, Ali Moradi

**Affiliations:** 1 Orthopedics Research Center, Mashhad University of Medical Sciences, Mashhad, 91388-13944 Iran; 2 Department of Community Medicine and Public Health, Ghaem Hospital, Mashhad University of Medical Sciences, Mashhad, Iran

**Keywords:** Cervical radiculopathy, Psychology, Carpal tunnel syndrome, Disability

## Abstract

**Background:**

Regarding musculoskeletal conditions, patient’s psychological distress, are shown to be associated with higher disability. Cervical radiculopathy (CR) and carpal tunnel syndrome (CTS), are two conditions caused by entrapment of cervical nerve roots and carpal median nerve, respectively. This study aims to investigate the association of psychological factors including depression, anxiety, and pain catastrophizing, with measures of upper limb patient-reported and performance-based disability, in patients with CR, and compare the obtained results with our similar study on CTS.

**Methods:**

In a cross-sectional study, we recruited 92 patients with CR, and investigated their disability level using patient-reported questionnaires (Quick Disabilities of the Arm, Shoulder and Hand (DASH) and pain Likert Scale) and by measuring grip and pinch strength. We also assessed their psychological status with Hospital Anxiety and Depression Scale questionnaire for depression (HADS-D) and anxiety (HADS-A) and also Pain Catastrophizing Scale (PCS) tools. We performed correlational coefficient analysis between disability and psychological scores and regression analysis of dependent variables (Pain, DASH, grip and pinch scores) and independent (psychological) variables. Finally, Z observed value was calculated to compare correlational coefficients between two diseases of CTS and CR.

**Results:**

The results of the correlational coefficient analysis indicate that all three HADS-A, HADS-D and PCS scores correlated with DASH score (*r* = 0.49, 0.37, 0.38 for HADS-A, HADS-D and PCS, respectively; *p* < 0.001 for all three). HADS-A also significantly correlated with VAS pain score (*r* = 0.41, *P* < 0.001) and grip strength (*r* = − 0.25, *P* = 0.016). Linear regression analysis revealed that anxiety has a notable value for DASH and VAS pain scores as well as grip strength. Fisher’s r correlation coefficient to z transformation, revealed that there was no difference between two diseases of CTS and CR in terms of the resulted r coefficients from correlational coefficient analysis between disability and psychological distress.

**Conclusion:**

It is concluded that psychological disorders are associated with disability in CR patients, with anxiety also correlating with objective disability parameter of grip strength. Finally, both CTS and CR patients’ disabilities associate with anxiety, depression, and catastrophysing thinking in a similar manner.

**Level of evidence:**

Level IV (cross-sectional study).

**Supplementary Information:**

The online version contains supplementary material available at 10.1186/s12891-022-05593-2.

## Introduction

### Background

Cervical Radiculopathy (CR) is a common clinical condition [1], resulting from compression of cervical nerve roots, and responsible for neck and upper limb symptoms and disability [2]. The incidence rate has been estimated as 83.2 per 100,000 people, with the highest rate in the fifth decade [3, 4]. It has been shown that psychological factors, which take account of patient’s psychological and social behaviours, are associated with higher neck disability in patients with CR [1, 5]. Women have higher incidence of CR as well as psychological distress conditions [6].

Carpal tunnel syndrome (CTS) is a common compressive median neuropathy, as the nerve transverses through the wrist. Radicular discomfort, paresthesia, and numbness may be felt distal (hand) or proximal (arm) to the wrist in the median nerve territory [7]. Women are more likely to be affected by the risk factors, which include high force and highly repetitive wrist activities [8], obesity, diabetes, pregnancy, and others (9.2% incidence rate in women vs 6% in men) [9, 10].

### Rationale

Following a similar study on the correlation between disability and psychological factors in patients with Carpal Tunnel Syndrome (CTS) [11], the current study attempted to examine the effect of same psychological factors on disability scores in patients with CR, ultimately comparing their results. CR and CTS can occur concomitantly [12, 13]. These two conditions may also be difficult to differentiate at times in clinical practice as may cause similar symptoms including radiating pain and tingling [6]. Patient-reported outcome measures and patient’s own perspective of their general health state have recently become an appropriate tool both in making the decision about treatment approach and evaluation of its effectiveness [5, 14]. The hypothesis here is that patient-reported disability may be correlated to psychological factors which might be beneficial to be addressed prior to final decision for surgical intervention. That being said, knowledge is limited about specific mental distress factors which contribute to the disability in patients with CR. It also remains to be answered whether psychological factors are associated with disability parameters of grip and pinch strength which are not patient-reported [15–17]. This study is therefore concerned with considering psychological factors when assessing patients with upper limb symptoms suggestive of CR, as confounders on disability score.

### Questions

We therefore asked: What are the relationships among validated scores for (1) depression, (2) anxiety, (3) pain catastrophizing and measures of upper limb patient-reported and performance-based disability, in patients with CR? What is the (4) simultaneous association of psychological factors on disability in patients with CR? (5) How are the results of this study, with regard to the correlation between patients’ disability and psychological factors, comparable to CTS in a similar study [11]? This last question was a comparison between the results of this study and a similar study by our research team.

## Methods

### Settings and approval

This cross-sectional study was carried out in an urban tertiary hospital in 2019. Prior to commencing the study, ethical approval was obtained from the related research ethics committee (the ethics code is removed due to issues with blinding), and the study was conducted in accordance with the ethical standards in the 1964 Declaration of Helsinki.

**Fig. 1:**
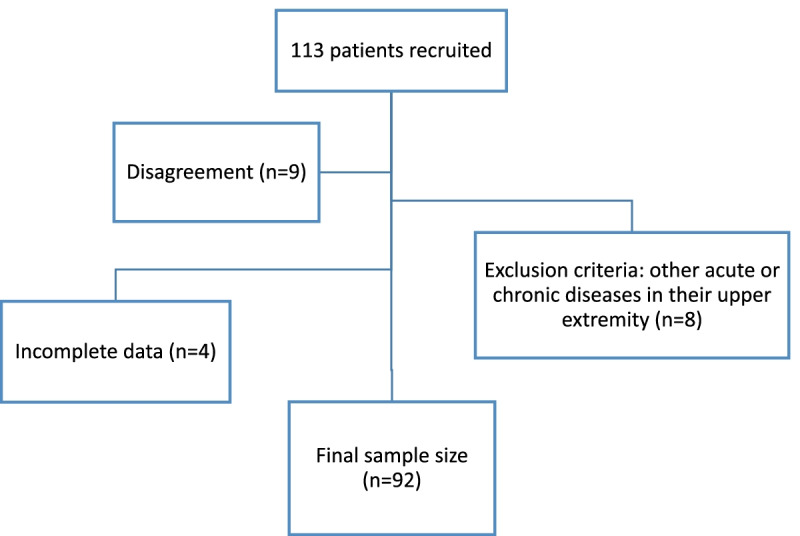
Excluded patients’ flow chart

We recruited from patients who were clinically diagnosed as having CR [18], and subsequently referred to our EMG-NCS examination at our tertiary level hospital rehabilitation facility. Our rehabilitation and physical medicine specialist (Dr. A.A.) visited patients again at the center and confirmed the diagnosis in 113 of them, who were then recruited using consecutive convenience sampling method. The Inclusion criteria were: age more than 18, radicular neck and arm pain with a corresponding one cervical nerve root, paresthesia, reduced tendon reflexes, or motor weakness corresponding to a specific dermatome. All patients received an explanation of the project and were provided with and signed written informed consent forms before being enrolled. 92% (104 of 113) of patients with CR agreed to participate in the study. Patients were excluded from the sample if they had other acute or chronic diseases in their upper extremity including related congenital conditions, prior surgery due to a condition other than CR, acute or complicated fracture and other types of neuropathy, or were pregnant. Accordingly, 7% (8 of 113) of patients were excluded. A further 3.5% (4 of 113) of patients were lost due to incomplete data, leaving 92 patients for final analysis (Fig. 1).

### Study design

We created the data sheet where we gathered demographic data including patients’ personal (age, sex, education) and medical (symptom duration, dominant hand, involved side, history of prior medical comorbidities) information. We also assigned each patient a code in order to protect their privacy and to prevent exposure of their names. On arrival at the centre, patients’ upper limbs were first assessed using both bilateral EMG results and dynamometer in order to examine grip and pinch force. For the purpose of clinically determining participants’ limb disability, patients were then asked to fill out the Quick DASH questionnaire. Furthermore, the questionnaires of HADS, PCS and Likert pain score were filled out in the same visit session. In case of confirmed bilateral radiculopathy, the more involved limb according to EMG-NCS result was considered for further analysis.

### Tools

#### EMG-NCS

We used a Dantec Counterpoint or Keypoint Electro-myographic machine for this research. In SNCV, filter settings ranged from 20 to 2000 Hz, but in MNCV, filter settings ranged from 20 to 10,000 Hz. The limbs were adequately warmed to keep the skin between 32 and 34 degrees Celsius. The deltoid (C5-C6), biceps (C5-C6), flexor carpi radialis (C6-C7), extensor digitorum (C6-C8), dorsal interosseous (C8-T1), and opponense pollicis (C8-T1) electromyography, were the least muscles studied in EMG. Abnormal EMG is defined as neurogen findings in a specific myotome consistent with cervical root lesion. Almost all NCS studies (F wave) were normal.

#### Quick Disabilities of the Arm, Shoulder and Hand (DASH)

Eleven-item quick DASH questionnaire is an abbreviated version of original DASH [19]. The questionnaire quantifies patients’ physical disability and symptoms in musculoskeletal disorders of the upper limb. The first six questions measure patients’ ability for doing different activities, and the final five questions deal with sleep quality, social and regular daily activities, pain severity and tingling [20]. Each item has a 5-point scale where the patient can select the appropriate number corresponding to his/her function level. Based on item scores, scale scores are calculated ranging from 0 (no disability) to 100 (most severe disability) [20] . We used the translated and validated version of the questionnaire [21]. The minimum clinically important differences (MCIDs) is determined as 15.91 points using distribution- and anchor-based approaches [22].

$$\mathrm{quick}\ \mathrm{DASH}\ \mathrm{Score}=\left(\left[\frac{\mathrm{sum}\ \mathrm{of}\ \mathrm{n}\ \mathrm{responses}}{\mathrm{n}}\right]-1\right)\ast 25$$

#### Pain severity

In order to quantify the severity of pain, 11 point Likert is used as a simple scale which scores pain severity from 0 (no symptom) to 10 (worst symptom) on a 11-point basis [23].

#### Grip and pinch dynamometer

A hand dynamometer is an apparatus for determining isometric grip force (hand grip strength). By using a hydraulic hand dynamometer (Jamar, Jackson, MI, USA), we measured patients’ isometric grip force (hand grip strength) in kilograms. Patients squeezed the dynamometer three times in Jamar position 2 [24], applying all of their force. Using Lafayette Hydraulic Pinch Gauge (Lafayette Company, Indiana, USA), we measured the palmar pinch. The gauge was placed between the pad of the thumb (superior to gauge), and the pads of all four fingers (inferior to gauge), while testing, and the patient was advised to pinch as forceful as possible. All measures performed on the affected limb side based on clinical symptoms.

#### Hospital Anxiety and Depression Scale (HADS)

To quantify and measure depression and anxiety of outpatients in clinics, the HADS questionnaire was designed with two subscales. Both subscales of HADS-D (depression) and HADS-A (anxiety) consist of seven items, and patients can respond on a scale of zero to three where zero designates no depression or anxiety and three designates their highest level. In this study, a translated version of this questionnaire which has been validated was used. To interpret patient scores, total subscale scores of 0–7 are considered as Normal, 8–10 as Borderline Abnormal (borderline case) and 11–21 as Abnormal (case). The MCIDs is 1.7 points from the distribution-based, anchor-based, and Delphi-based findings [25].

#### Pain Catastrophizing Scale (PCS)

As an attempt to evaluate the mental state of patients in pain, the 13-item instrument PCS was designed with three subscales assessing rumination, magnification and helplessness. Taking this test, patients need to answer how much they have experienced each of the 13 thoughts and emotions while being in pain. On its 5-point scale answer sheet, they can choose among 0 (not at all) to 4 (all the time), and their total scores may range from 0 to 52 [26]. In this study, a translated and validated version of the questionnaire is used [27].

### Statistical analysis

All analyses were carried out using SPSS software (version 22). The scores of questionnaires, DASH, HADS, PCS and VAS, recorded from a total of 100 in order to make analysis and interpretation easier.

We have reported the quantitative data as mean (standard deviation) for normally distributed data and as median (min - max) for non-normally distributed data. Our primary study goal was to evaluate the correlation of depression, anxiety and pain catastrophizing with affected limb disability. To achieve this, bivariate correlational analysis between psychological (HADS and PCS scores) and disability (grip and pinch strength, DASH and pain scores) parameters was performed using Spearman test. Then, the related *P value* and r coefficient were calculated. Adopting the linear regression model, it was possible to identify associated psychological factors and score of DASH, pain Likert, grip and pinch strength. *P value* significance level was set at 0.05. The secondary goal was to compare the correlational results between patients with CR and CTS (using our previous study results). To achieve this, Fisher’s r correlation coefficient to z transformation was conducted and z values (z1 and z2) that correspond to the correlation coefficients (r1 and r2) were calculated, and finally the comparison was conducted based on achieved Z observed value.

## Results

### Descriptive data

Ninety-two patients with confirmed CR were studied, 77 (83%) of whom were women. Table 1 presents some of the main characteristics of the population. Hypertension was the most common medical comorbidity (*N* = 11, 12%), and depression was the most common psychological disorder (*N* = 15, 16%). Of note, there were patients with more than one medical comorbidity, psychological comorbidity or previous received treatment, which is why in the related table sections, the sum of subsections are more than 100% (Table 1).

**Table 1:** Patients’ demographic data

Variable	CR (92) Present study	CTS (70) Prior study
Sex *number (%)*		
Women	77 (83.7)	62 (89)
Men	15 (16.3)	8 (11)
Age (year) *mean ± SD*	41.5 ± 11.1	47.1 ± 11.9
Education (year) *median (min-max)*	9.1 (0–25)	5 (0–18)
Occupation *number (%)*		
Housekeeper	55 (60)	52 (74)
Employed	16 (17)	14 (20)
Student	4 (4.34)	0 (0)
Others	17 (18)	4 (6)
Hand side involvement *number (%)*		
Right	35 (38)	14 (20)
Left	31 (33)	12 (17)
Bilateral	26 (28)	44 (63)
Disease duration (month) *median (min-max)*	22.4 (0.25–240)	6 (0–72)
Medical comorbidities *number (%)*		
No comorbidity	78 (84.8)	48 (69)
At least one comorbidity	14 (15)	22 (31)
a. Diabetes	3 (3)	11 (16)
b. Hypertension	11 (12)	15 (21)
c. Thyroid disease	1 (1)	0 (0)
Psychological background comorbidities^a^ *number (%)*		
No comorbidity	72 (78)	48 (69)
At least one comorbidity	20 (22)	22 (31)
a. Anxiety	7 (8)	9 (13)
b. Depression	15 (16)	10 (14)
c. Obsessive	3 (3)	4 (6)
d. Schizophrenia	1 (1)	1 (1)
Previous treatment for CR^a^ *number (%)*		
No previous treatment	68 (74)	39 (56)
At least one previous treatment	24 (26)	31 (44)
a. Medicine	8 (8.7)	31 (44)
b. Physiotherapy	11 (12)	
c. Steroid injection	10 (10.9)	7 (10)
d. Splint	2 (2.2)	1 (1)
e. Surgery	1 (1.1)	0
Questionnaire scores *mean ± SD*		
DASH score	50.2 ± 23.1	32.7 ± 8.8
PCS	28.9 ± 10.4	27.5 ± 12.0
HADS-A	9.5 ± 4.8	10.6 ± 5.1
HADS-D	8.2 ± 4.2	9.3 ± 4.2
Pain scores	64.6 ± 27.0	59.5 ± 26.4
Force (kg) *mean ± SD*		
Grip strength	15.0 ± 11.3	16.0 ± 8.8
Pinch strength	4.1 ± 2.6	4.2 ± 2.0
EMG-NCS *number (%)*		
Normal	10 (11)	0 (0)
Abnormal (CR)	82 (89)	
Abnormal (CTS)		70 (100)

*CR* cervical radiculopathy, *DASH* Quick Disabilities of the Arm, Shoulder and Hand, *HADS-A* Hospital Anxiety and Depression Scale-Anxiety, *HADS-D* Hospital Anxiety and Depression Scale-Depression, *PCS* Pain Catastrophysing Scale

^a^Based on patients’ medical document

### Association of Scores for depression and measures of hand disability

As HADS-D scores increased, so did DASH score (correlation coefficient = 0.34 [*p* = 0.001]). There was no relationship between the HADS-D and other studied variables in the analysis (Table 2).

**Table 2:** The correlation coefficient of HADS and PCS questionnaires with disability, pain, grip and pinch in patients with CR

	HADS-A		HADS-D		PCS	
	***P*** value	r coefficient	***P*** value	r coefficient	***P*** value	r coefficient
**Pain Likert score**^**a**^	**< 0.001**	0.417	0.138	0.156	**< 0.001**	0.363
**DASH score**^**b**^	**< 0.001**	0.473	**0.001**	0.340	**< 0.001**	0.411
**Grip strength**^**a**^	**0.016**	−0.251	0.215	−0.130	0.15	−0.151
**Pinch strength**^**a**^	0.513	−0.080	0.32	−0.121	0.642	−0.057

^a^Spearman correlation test

^b^Pearson correlation test

### Association of anxiety and hand disability

As HADS-A scores increased, so did DASH score (correlation coefficient = 0.47 [*p* < 0.001]), pain score (correlation coefficient = 0.41 [*p* < 0.001]), and grip force (correlation coefficient = − 0.25 [*p* = 0.016]) (Table 2). Every one-unit increase in HADS-A score led to 1.48 and 2.11 unit increase in DASH and pain score, respectively and 0.67 kg unit decrease in grip strength (Table 3).

**Table 3:** Parameter Estimates of the Linear Regression Model of DASH, Likert pain, and grip scores (dependent/response variable) against HADS and PCS scores in patients with CR

Parameter		R Square	Model ***P*** value	Unstandardized Coefficient B (Std. Error)	***P*** value
**DASH score**	HADS Anxiety	0.273	< 0.001	1.48 (0.53)	**0.007**
	HADS Depression			0.60 (0.57)	0.29
	PCS			0.46 (0.23)	0.052
**Pain Likert score**	HADS Anxiety	0.234	< 0.001	2.11 (0.64)	**< 0.001**
	HADS Depression			_0.25 (0.68)	0.71
	PCS			0.47 (0.28)	0.09
**Grip strength**	HADS Anxiety	0.089	0.016	−0.66 (0.27)	**0.018**
	PCS			−0.029 (0.12)	0.819

### Association of pain catastrophizing and hand disability

As PCS scores increased, so did DASH score (correlation coefficient = 0.41 [*p* < 0.001]) and pain score (correlation coefficient = 0.38 [*p* < 0.001]) (Table 2).

In general, 27.3% of the variance in DASH score was predicted from HADS-A, HADS-D and PCS scores (R Square = 0.273). Regression analysis shows that using HADS-A, HADS-D and PCS as three predictors was significantly better than prediction without them and using only the mean in the model (*P* value < 0.001) (Table 3).

### Comparison of CR and CTS regarding correlation of disability and psychological parameters

**Fig. 2:**
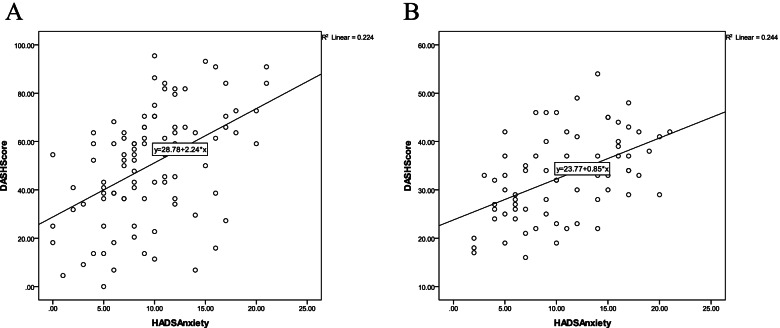
The graphs showing DASH-Anxiety scores correlation pattern in (**A**) CR and (**B**) CTS

We observed no difference between two conditions of CTS and CR r coefficients in terms of the correlation between disease disability scores and psychological parameters (Table 4, Fig. 2).

**Table 4:** Comparison between CTS and CR regarding r coefficients

Variable	r1 (CTS)	r2 (CR)	z1 (CTS)	z2 (CR)	N1 (CTS)	N2 (CR)	Z observed
**Pain/HADS-A**	0.21	0.41	0.12	0.29	70	92	−1.05
**Pain/PCS**	0.44	0.36	0.33	0.24	70	92	0.53
**DASH/HADS-A**	0.5	0.49	0.41	0.39	70	92	0.09
**DASH/HADS-D**	0.42	0.37	0.3	0.25	70	92	0.32
**DASH/PCS**	0.53	0.38	0.45	0.26	70	92	1.19
**Grip/HADS-A**	−0.15	− 0.25	− 0.07	−0.12	70	92	0.27

Table rows: The CR and CTS disability and psychological variables which were revealed to be correlated in present and prior CTS studies, included in this comparison

Table columns: r: r correlation coefficient; z: Z score (Fisher’s r to z transformation, is done so that the z scores can be compared and analyzed for statistical significance by determining the observed z test statistic); Z observed: with Z observed at a set level of significance, statistical significance can be assessed

Level of significance set at 0.05, which indicates that the critical value is ±1.96, all of our Z observed fall into the accepted region and are within the critical value; thus, not statistically significant

Level of significance set at 0.05, which indicates that the critical value is ±1.96, all of our Z observed fall into the accepted region and are within the critical value; thus, not statistically significant.

## Discussion

### Background

The severity of disability reported by patients with CR, has been thought to be affected by psychological status [2]. Since patient-reported tool has been considered in treatment approach [14], we get interested to evaluate it’s association with mental distress to see if we should pay more attention to the patients’ psychological status. This study thus set out with the aim of assessing the importance of psychological factors in chronic CR patients’ disability. The most obvious finding to emerge from the analysis is that anxiety had the most significant correlations with disability parameters in CR including DASH score, pain intensity and grip strength. Our study highlighted the importance of psychological distress to be considered in patients with CR as a conservative treatment before deciding for invasive surgical intervention.

### Limitations

The patients consisted of more than 50% with housekeepers, more even distribution of patients’ occupation seems to be ideal. BMI is a missed variable in our demographic data. The maximum score would be a better representation of the variable than the average of three dynamometry measures for grip and pinch strength collected and assessed in our study.

### Discussion of key findings

We found that scores for all three mental distress parameters (anxiety, depression and pain catastrophizing) had positive correlation with patient-reported disability (DASH) in patients with cervical radiculopathy. It has previously been found that disability in patients with CTS has similar association with mental distress. This finding thus provides support for examination and treatment of mental distress signs in patients with marked disability. As Nikola et al. concluded, this relationship maybe due to the correlation between pressure pain threshold (PPT) measured by the algometer and anxiety/depression, using the same questionnaires as ours (HADS) [28]. Consistent with our results, Conradie et al. also investigates patients with chronic CR and states that functional abilities in acute CR correlate with anxiety more than depression level [1].

There are several studies indicating that psychological factors are also important predictors in surgical treatment outcome or post operation disability in CR patients [2, 29–31]. On the contrary, Doi et al. shows that improvements in health-related quality of life outcomes post operatively is independent of the presence of preoperative depression or anxiety in patients with cervical compressive myelopathy [32]. The magnitude of improvement post-operatively is shown to be similar among distressed and non-distressed patients in orthopaedic surgery since pain relief can improve mental health itself [33]. Our hypothesis is that post-operative improvements may also be relatively due to the mental effect of undergoing surgical intervention and surgical placebo effect which has to be considered while interpreting results of surgeries with patient-reported outcomes [34].

As an interesting finding, we also found a modest correlation between anxiety and grip strength as well as anxiety also values for grip force variance. Another study has also found a similar negative correlation between depression/anxiety and hand strength but in stroke patients [35]. Previous studies mainly conclude that motor function is associated with psychological distress far less than disability measured using self-reported health-status questionnaires [36, 37]. However, our results suggest that anxiety as a variable of psychological distress also correlates with grip strength as a variable of motor function. Albeit, we should consider the mentioned studies were conducted on less severe upper limb conditions. This finding further supports the consideration of a psychosocial model, besides the main treatments, when managing patients who seem to be experiencing mental distress symptoms.

We observed that patient-reported disability scores in patients with either CTS or CR similarly associate with psychological parameters, and there is no notable difference in two conditions’ r coefficients. Anxiety seems to be the parameter which correlates stronger with disability in patients with either of these diseases. The only difference between the results of these two studies is the negative correlation of anxiety with grip strength in patients with CR. While psychological factors were only associated with patient-reported disability scores in patients with CTS [11], they also affected objective evaluations in patients with CR. This last finding emphasizes the important role of psychological distress on evaluation outcomes as it changes the results of objective measurements as well. Therefore, we also recommend considering psychological elements before any decision for surgical intervention as a first complementary step in treating patients with CR.

## Conclusion

The results of this investigation show that anxiety correlates with disability parameters of DASH, pain, and grip scores in patients with CR. In addition, the pattern of correlation between psychological and disability factors are almost similar between patients with CR and CTS.

Based on the present results, we cannot conclude if mood disorders result in more disability or disability leads to depression and anxiety. This remains to be determined in further longitudinal studies. Furthermore, it is also unclear whether psychological intervention can improve pain and functional ability post-operatively.

## Supplementary Information

**Additional file 1.**

## Data Availability

Correspondence and requests for materials should be addressed to almor0012@gmail.com.
